# Assessment of Cognitive Functions Among Remitted Patients of Schizophrenia and Bipolar Disorder: A Comparative Study

**DOI:** 10.7759/cureus.64296

**Published:** 2024-07-11

**Authors:** Selvameenatchi R, Ramya Rachel Jetty, Aruna Kaki, Suresh Kumar Gunapalli, Prasanna Kumar N, Arul Saravanan R

**Affiliations:** 1 Psychiatry, Sri Ramaswamy Memorial (SRM) Medical College Hospital and Research Center, Chennai, IND; 2 Psychiatry, Sri Ramaswamy Memorial (SRM) Medical College Hospital and Research Center, SRM Institute of Science and Technology, Chennai, IND; 3 Psychiatry, Andhra Medical College, Visakhapatnam, IND

**Keywords:** india, neuropsychological test, cognitive functions, bipolar disorder, schizophrenia

## Abstract

Introduction

Bipolar disorder and schizophrenia exhibit different patterns of cognitive impairment, with schizophrenia demonstrating more profound deficiencies in verbal memory and bipolar disorder in social cognition. Understanding these patterns may guide the development of interventions to enhance cognition in these disorders.

Aim

This study aims to assess and compare the cognitive abilities of persons diagnosed with bipolar illness and schizophrenia.

Methodology

A facility-based cross-sectional study was done from December 2016 to June 2017 among 30 schizophrenia and 30 bipolar disorder patients aged 18-45 years, in remission selected after screening through Hamilton Depression Rating Scale (HDRS), Young Mania Rating Scale (YMRS), or Positive and Negative Syndrome Scale (PANSS). Exclusions included schizoaffective disorder, systemic illness, brain/neurological conditions, and substance abuse. After collecting the baseline demographic and clinical profile of the selected patients, the cognitive domains were assessed such as attention (digit span), verbal memory (Rey's Auditory Verbal Learning Test (RAVLT)), visual memory (Rey Complex Figure), verbal fluency (Animal Naming), and executive functions (Stroop and Trail Making). The data was analyzed using the IBM SPSS Statistics for Windows, Version 16 (Released 2007; IBM Corp., Armonk, New York, United States) using standard descriptive and inferential statistics.

Results

Sociodemographic and clinical characteristics were largely similar between groups. Schizophrenia patients showed poorer attention, working memory, and visual attention/task-switching compared to bipolar patients. Bipolar patients demonstrated relatively preserved abilities in these domains but exhibited more impairments in visual and verbal memory. Distinct patterns highlight unique neurobiological underpinnings, showing association of more generalized cognitive deficits in schizophrenia and more localized impairments in memory functions in bipolar disorder.

Conclusion

The study findings explain these disorders' unique neurobiological mechanisms and may help develop targeted cognitive remediation and pharmacological interventions to improve functional outcomes and quality of life.

## Introduction

Bipolar disorder and schizophrenia are discrete but debilitating psychiatric disorders that may profoundly affect an individual's cognitive abilities. Both illnesses often exhibit cognitive deficits that impact many areas including attention, processing speed, memory, and executive functioning [1,2]. Cognitive functions include attention, processing speed, memory, executive functioning, and social cognition.

Bipolar disorder, which involves alternating bouts of mania and depression, is known to affect cognitive performance both during acute episodes and in euthymic states [3]. People diagnosed with bipolar disease often have impairments in verbal memory, attention, and executive functioning, which may continue to exist even during times of remission [4]. The cognitive deficits may significantly affect one's capacity to operate daily, find employment, and overall quality of life.

However, schizophrenia is a long-lasting and incapacitating mental condition that is defined by the presence of positive symptoms and negative symptoms [5]. Cognitive impairments are a fundamental characteristic of schizophrenia, with extensive deficiencies seen in several areas, such as attention, working memory, processing speed ,verbal and visual learning, and executive functioning [2,6]. These cognitive impairments often occur before the appearance of psychotic symptoms and may contribute to the functional limitations encountered by persons with schizophrenia.

Although there may be some similarities in cognitive impairments between bipolar illness and schizophrenia, the differences in the magnitude and pattern of abnormalities may be attributed to the unique neurological mechanisms that underlie each disease. Deficits in social cognition and working memory are more severe in schizophrenia, but impairments in verbal memory and executive functioning may be more noticeable in bipolar illness [7].

Gaining a comprehensive understanding of the distinct cognitive profiles associated with bipolar illness and schizophrenia might provide useful insights into the fundamental neurobiological causes and possible targets for therapeutic interventions in these diseases. Researchers and clinicians may create personalized cognitive remediation techniques and psychosocial treatments to improve functional results and enhance the quality of life for patients by identifying the specific areas of cognitive strength and weakness associated with each condition [1].

Hence, our study aims to assess comprehensively and compare the cognitive abilities of persons diagnosed with bipolar illness and schizophrenia, utilizing standardized neuropsychological assessments and objective measures.

## Materials and methods

A cross-sectional study was conducted from December 2016 to June 2017 among bipolar and schizophrenia patients attending the outpatient and inpatient services of the Government Hospital for Mental Care in Visakhapatnam, South India. The study was done after obtaining ethical approval.

Those patients aged 18-45 years of both genders, able to give valid consent, and had at least eight years of formal education diagnosed with either bipolar or schizophrenia as per ICD-10 classification were screened for eligibility. Bipolar patients underwent screening using the Hamilton Depression Rating Scale (HDRS) [8] to detect depression and the Young Mania Rating Scale (YMRS) to evaluate manic symptoms [9]. Only bipolar patients who had an HDRS score of less than 8 and a YMRS score of less than 6 had been in remission for more than four months and were being treated with a single mood stabilizer were included in the research.

Furthermore, the Positive and Negative Syndrome Scale (PANSS) to assess the symptom severity in schizophrenia patients [10]. Only the patients who met the following criteria were included: a PANSS score below 60, a remission period of at least six months, and being on a single antipsychotic medication. The research excluded individuals with bipolar disorder or schizophrenia who had a diagnosed schizoaffective disease, evidence of a significant systemic illness, an organic brain or neurological condition, a history of drug misuse or dependency, or a history of electroconvulsive therapy within the preceding six months.

A total sum of 30 schizophrenia patients and 30 bipolar disorder patients satisfying the inclusion and exclusion criteria were finally enrolled sequentially for the study. Written informed consent of the participants was obtained prior to initiating the study. The neurocognitive assessment was made by a single qualified psychiatrist through a semi-structured interview schedule for all patients to avoid measurement bias. After collecting the baseline demographic and clinical profile of the selected patients, five varied cognitive domains were evaluated including attention, visual memory, verbal memory, verbal fluency, and executive functioning. To facilitate comparisons with other studies, the evaluation was made with a selection of available and widely used neuropsychological tests.

Attention was evaluated using the digit span test, which includes two tasks: digit forward and digit backward. The digit forward span test assesses the ability to recall and repeat a sequence of numbers in the correct order. Digit backward span necessitates the reversal of the sequence. Subpar performance, particularly in the backward task, suggests attention and working memory impairments often seen in individuals with bipolar illness and schizophrenia [11].

The Rey's Auditory Verbal Learning Test (RAVLT) was used to assess the verbal memory. The RAVLT assesses an individual's ability to acquire and retain verbal information presented in the task. The task comprises of remembering a list consisting of 15 words in five trials, which include interference and delayed recall trials. Scores evaluate cognitive aptitude as well as the impact of proactive and retroactive interference on memory retention and retrieval. It is beneficial for assessing memory impairments in conditions such as bipolar disorder and schizophrenia [12].

The assessment of visual memory was conducted using the Rey Complex Figure Test (RCFT). The RCFT evaluates visuospatial aptitudes, memory retention, and executive cognitive processes. The task entails replicating an intricate pattern, promptly retrieving it from memory, then remembering it again after a 20-30-minute interval. Scoring evaluates accuracy and placement of design elements [13].

The Animal Naming Test (ANT) was used to evaluate verbal fluency. The participants are required to list as many animals as they can within the timeframe of 60 seconds. The scoring is calculated by the total number of unique names created correctly. The test detects cognitive impairments in individuals with bipolar illness and schizophrenia that are associated with abnormalities in the frontal lobe [14].

The assessment of the executive functions was conducted using the Stroop Test and the Trail Making Test. The Stroop Test evaluates an individual's ability to focus on specific information, adapt to changing circumstances, and suppress irrelevant thoughts or actions. It necessitates identifying the ink color of written words when the phrase itself represents a contradictory color name. The presence of more mistakes and a decrease in speed suggest challenges that impede the automated process of reading words. Individuals with bipolar illness and schizophrenia are prone to frontal lobe/executive impairments [15]. The Trail Making Test assesses the cognitive abilities of visual scanning, sequencing, set-shifting, divided attention, and working memory, applicable for evaluating cognitive flexibility and control. The composition comprises two components. Part A: Connect the numerals that are circled in sequential sequence. Part B: Establish a connection between numbers and letters in an alternating sequence, following the order of 1-A-2-B, and so on. The time duration for the test was assessed using a digital clock [16].

The data obtained from the interview schedule was charted in MS Excel (Microsoft Corporation, Redmond, Washington, United States), and the IBM SPSS Statistics for Windows, Version 16 (Released 2007; IBM Corp., Armonk, New York, United States) was used for statistical analysis. The comparison of various characteristics for qualitative variables was done using chi-square test and for qualitative variables by independent t-test. A p-value of <0.05 was considered statistically significant with 95% confidence interval. Mean/standard deviation versus median/interquartile range were used to sum up the quantitative variables, while those of qualitative variables were summarized as frequency and percentage.

## Results

This study included a total of 60 patients, 30 each with schizophrenia and bipolar disorder who were in remission. The sociodemographic characteristics of both patient groups are explained in Table 1. The mean age in schizophrenia and bipolar patients were 32.57 (6.96) and 32.43 (5.32) years, respectively. Among the schizophrenia patients, the majority were males (60%), educated up to the 12th standard (60%), unmarried (60%), currently unemployed (87%), residing in rural areas (70%), and belonging to the lower middle/upper lower socioeconomic status (60%). On the contrary, the majority of bipolar disorder patients were males (70%), had a degree (53.3%), married (53.3%), employed currently (60%), residing in rural areas (77%), and belonging to the upper and lower middle socioeconomic status (67%). However, other than occupation, all other sociodemographic characteristics were similar. Table 2 shows the clinical characteristics of schizophrenia and bipolar disorder patients. The age of onset of mental illness was 22.74 (±5.30) and 24.30 (±9.86) years among the schizophrenia and bipolar disorder patients respectively. The duration of illness among schizophrenia patients was 3.67 (±1.12) years and among bipolar disorder patients was 3.40 (±1.28) years. Both groups of patients had an average of two admissions for their mental illness, respectively. The clinical characteristics were similar in both the schizophrenia and bipolar disorder patients.

**Table 1:** Distribution of the sociodemographic variables among the two patient groups (n = 60) *p-value by chi-square test; #: p-value by independent t-test

	Schizophrenia, n (%)	Bipolar, n (%)	p-value*
Age (years), mean (±SD)	32.57 (±6.96)	32.43 (±5.32)	0.870^#^
Gender			0.589
Male	18 (60%)	21 (70%)	
Female	12 (40%)	9 (30%)	
Education			0.306
9th std to 12th std	18 (60%)	14 (46.7%)	
>12 std	12 (40%)	16 (53.3%)	
Marital status			0.306
Unmarried	18 (60%)	14 (46.7%)	
Married	12 (40%)	16 (53.3%)	
Occupation			0.004
Unemployed	26 (86.7%)	12 (40%)	
Employed	4 (13.3%)	18 (60%)	
Domicile			0.771
Rural	21 (70%)	23 (76.7%)	
Urban	9 (30%)	7 (23.3%)	
Socioeconomic status			0.467
Upper and upper middle	7 (23.3%)	8 (26.7%)	
Lower middle	10 (33.3%)	12 (40%)	
Upper lower	8 (26.7%)	7 (23.3%)	
Lower	5 (16.7%)	3 (10%)	

**Table 2:** Comparison of clinical characteristics among the two patient groups (n = 60) *p-value by independent t-test; #: p-value by Mann-Whitney U test

Clinical ch aracteristics	Schizophrenia, mean (±SD)	Bipolar, mean (±SD)	p-value*
Age at onset (years)	22.74 (±5.30)	24.30 (±9.86)	0.496
Illness duration (years)	3.67 (±1.12)	3.40 (±1.28)	0.279
Number of admissions, median (IQR)	2 (1-3)	2 (1-4)	0.903^#^

The comparison of the attention and verbal fluency domains are described in Table 3. The digit span test revealed significantly low digit forward test time among schizophrenia patients 5.20 (±1.56) minutes versus 6.27 (±1.62) minutes among the bipolar patient group. The digit backward test time was similar. The verbal fluency test by ANT revealed similar timelines for both animals and fruits among the schizophrenia and bipolar disorder patients. Table 4 compares the memory domains among the two patient groups. Almost all the RAVLTs for verbal memory were significantly higher time among bipolar patients compared with schizophrenia patients except for Trail 4, Trail 5, List B, and the Commissions test. Similarly, the Rey-Osterieth diagram for visual memory revealed significantly higher time among bipolar patients except for the Copy (Rey O Copy) component. The comparison of the executive functions is revealed in Table 5. The Stroop color test revealed nil significant difference between the two patient groups. The trail making test revealed significantly higher time for schizophrenia patients in the Trail A test compared with the bipolar patients. There was nil significant difference in the Trail B test between the two groups.

**Table 3:** Comparison of attention and verbal fluency domains among the two patient groups (n = 60) *p-value by independent t-test

	Schizophrenia, mean (±SD)	Bipolar, mean (±SD)	p-value*
Digit span (time in minutes)			
Digit forward	5.20 (±1.56)	6.27 (±1.62)	0.012
Digit backward	2.90(±1.32)	3.27(±1.76)	0.365
Animal Naming Test (number of names in 60 seconds)			
Animals	9.20 (±2.56)	9.87 (±3.08)	0.366
Fruits	8.77 (±2.13)	9.20 (±2.09)	0.430

**Table 4:** Comparison of memory domains among the two patient groups (n = 60) *p-value by independent t-test

	Schizophrenia, mean (±SD)	Bipolar, mean (±SD)	p-value*
Rey Auditory Verbal Learning Test (number of names)			
Trail A1	4.50 (±2.13)	5.67 (±2.04)	0.034
Trail A2	5.40 (±2.11)	7.33 (±2.43)	0.002
Trail A3	6.77 (±2.08)	8.30 (±3.22)	0.032
Trail A4	7.53 (±2.45)	8.30 (±3.02)	0.284
Trail A5	8.40 (±2.09)	9.53 (±3.01)	0.094
List B	4.90 (±2.11)	4.40 (±1.98)	0.347
Immediate recall A (IR A)	6.87 (±2.49)	7.50 (±2.89)	0.367
Delayed recall A (DR A)	4.60 (±2.92)	6.90 (±3.43)	0.007
Hits (H)	10.20 (±2.54)	12.23 (±2.37)	0.002
Omissions (O)	4.80 (±2.54)	2.77 (±2.37)	0.002
Commissions (C)	0.67 (±1.06)	1.03 (±1.43)	0.263
Rey-Osterrieth diagram (scores)			
Copy (Rey O Copy)	33.93 (±2.63)	32.73 (±6.68)	0.364
Immediate recall (Rey O IR)	14.07 (±5.43)	23.73 (±9.32)	<0.001
Delayed recall (Rey O DR)	11.33 (±4.92)	22.10 (±9.99)	<0.001

**Table 5:** Comparison of executive functions among the two patient groups (n = 60) *p-value by independent t-test

	Schizophrenia, mean (±SD)	Bipolar, mean (±SD)	p-value*
Stroop Color Test (time in seconds)			
Stroop 1	74.70 (±14.15)	83.53 (±33.634)	0.190
Stroop 1 (error)	0.33 (±0.66)	0.27 (±0.828)	0.732
Stroop 2	136.23 (±28.76)	139.53 (±49.963)	0.755
Stroop 2 (error)	1.90 (±2.37)	2.23 (±4.987)	0.742
Trail Making Test (time in seconds)			
Trail A	121.93 (±51.99)	90.00 (±27.26)	0.004
Trail B	279.23 (±135.92)	230.70 (±67.27)	0.085

## Discussion

The current study offers an extensive evaluation of cognitive functioning in patients with schizophrenia and bipolar disorder, demonstrating distinct patterns of impairments across a variety of cognitive domains. These findings enhance understanding of the distinctive neurobiological mechanisms that underlie these disorders and have substantial implications for clinical practice and future research.

In comparison to patients with bipolar disorder, patients with schizophrenia demonstrated substantially lower performance on the digit forward test, suggesting impaired attention and working memory capacity. This finding is in accordance with prior research indicating that schizophrenia patients exhibit more severe impairments in their attention and working memory [1,17,18]. Nevertheless, the digit backward test and the verbal fluency task demonstrated comparable impairments in these domains for both groups. The relatively preserved verbal fluency in both disorders may be attributed to the regulation of this cognitive function by contiguous brain regions, such as the prefrontal cortex and temporal lobe [19].

The research demonstrated substantial disparities in memory performance between the two groups. In comparison to individuals with schizophrenia, patients with bipolar disorder exhibited superior performance on nearly all components of the RAVLT. This suggests that they possess superior verbal memory abilities. The result is in accordance with prior research that has documented more severe verbal memory impairments in schizophrenia [4]. The differential involvement of brain regions that are essential for memory functioning, such as the hippocampus and associated structures, may be the cause of the distinct patterns of verbal memory impairments observed in these disorders [5]. In the same vein, patients with bipolar disorder demonstrated superior performance on the Rey-Osterrieth Complex Figure Test, indicating that their visual memory was relatively well-preserved compared with that of schizophrenia patients. This discovery is consistent with prior research that has suggested that schizophrenia patients exhibit more obvious visual memory deficits [20].

The assessment of executive functions revealed mixed findings. Although both groups showed similar performance on the Stroop Color Test, which measures cognitive flexibility and inhibitory control, individuals with schizophrenia took significantly more time to finish Trail A of the Trail Making Test. This finding indicates that individuals with schizophrenia have diminished visual attention and task-switching skills in comparison to those with bipolar disorder [7]. Nevertheless, there was no notable disparity observed in the results for Trail B, which evaluates intricate executive functions that involve set-shifting and working memory. The relatively preserved performance on certain executive function tasks in bipolar disorder may be attributable to the less severe impairments in frontal lobe functioning compared to schizophrenia [6].

The distinct patterns of cognitive impairments observed in this study highlight the unique neurobiological mechanisms underlying bipolar disorder and schizophrenia. Schizophrenia is marked by more profound structural and functional abnormalities in brain regions critically those involved in attention, working memory, and executive functioning, such as the prefrontal cortex, parietal cortex, and associated networks [21]. In contrast, bipolar disorder is associated with more localized impairments in verbal memory and executive functions, potentially linked to dysfunction in the hippocampus, amygdala, and frontal lobe regions [22]. Identifying specific cognitive strengths and weaknesses can assist in developing cognitive remediation strategies and psychosocial interventions. There is extensive research on cognitive functions in symptomatic patients of schizophrenia and bipolar patients when compared with those of remitted patients; this increases the strength of the study. The current study is also subject to certain limitations like a relatively small sample size, the cross-sectional nature and lack of control group. Further longitudinal research involving larger sample numbers are necessary to further confirm and build upon these findings.

## Conclusions

In this study, patients with schizophrenia exhibited more profound deficits in attention and working memory, while those with bipolar disorder demonstrated relatively preserved cognitive abilities in these domains. However, both groups exhibited impairments in verbal and visual memory, with more severe deficits observed in schizophrenia. The evaluation of executive functions revealed mixed results, with schizophrenia patients exhibiting impaired visual attention and task-switching abilities, while performance on other executive function tasks was comparable between the groups. These findings help explain the unique neurobiological mechanisms of these disorders and may help develop targeted cognitive remediation and pharmacological interventions to improve functional outcomes and quality of life.
